# Appraising the Physical Activity Levels of Saudis with Physical Disabilities: Effects of Disability Type, Mobility Assistive Devices, and Demographic Factors

**DOI:** 10.3390/healthcare12090937

**Published:** 2024-05-02

**Authors:** Mohamed A. Said, Majed M. Alhumaid

**Affiliations:** 1 Department of Physical Education, College of Education, King Faisal University, Al-Ahsa 31982, Saudi Arabia; 2 Higher Institute of Sport and Physical Education of Kef, University of Jandouba, Kef 7100, Tunisia

**Keywords:** physical activity, physical disability, type of disability, mobility assistive devices, marital status, education, occupation

## Abstract

Physical activity (PA) has numerous health benefits for individuals with physical disabilities (IWPD). However, it is common for activity levels to fall below the suggested limits. This study aimed to evaluate the prevalence, pattern, and levels of PA among IWPD in Saudi Arabia. It also investigated the effects of individuals’ type of disability, mobility assistive devices, and demographic features on PA levels. Data were collected from 238 participants, mostly male (62.2%), aged 39.76 ± 12.19 years. Among them, 19.3% had spinal conditions, 14.7% had progressive muscular dystrophy, 15.1% had multiple sclerosis, 17.6% had cerebral palsy, 16.4% had poliomyelitis, and 16.8% had limb or foot amputations. The participants were assessed using the Arabic version of the Physical Activity Scale for Individuals with Physical Disabilities (PASIPD-AR). The results showed that 62.6% (64.9% of males and 58.9% of females) met the minimum PA guidelines specified by the WHO. The average PASIPD-AR score was 10.33 ± 10.67 MET-hours/day, indicating lower PA levels, and 8.4% of individuals did not participate in any form of PA. Significant discrepancies were detected in disability type and mobility assistive device use after age adjustment. Marital status, education, and occupation greatly affected PA components. Greater attention should be paid to promoting an active lifestyle among IWPD in Saudi Arabia.

## 1. Introduction

A physical disability is a significant and enduring restriction that impacts an individual’s ability to move, perform physical tasks, sustain activity, or demonstrate agility. It significantly impairs their capacity to perform some routine tasks [1], such as lifting items or getting dressed, which become increasingly challenging and time-consuming [2]. Individuals with physical disabilities (IWPD) may face challenges accessing the physical environment, safely using equipment and facilities, engaging in learning tasks and evaluations, and performing practical activities [3]. It has also been reported that IWPD have greater difficulties participating in society because they suffer a high level of social exclusion, including in education or participation in different social activities [4]. This situation means that IWPD are subject to the stereotypes, stigmas, and prejudices established by society.

Physical disabilities include many disorders such as spinal disease (SD), cerebral palsy (CP), stroke, multiple sclerosis (MS), progressive muscular dystrophy (PMD), poliomyelitis, arthritis, and amputation. Mobility limitations exhibit variability based on the specific disability, gender, age, and other relevant factors. For instance, a child afflicted with muscular dystrophy, confined to a wheelchair, and lacking motor control in their lower limbs may nevertheless possess the ability to utilize their upper limbs autonomously. However, they may still require assistance in areas such as movement and cleanliness. Another youngster with CP may possess the ability to navigate freely, although they may encounter greater challenges when it comes to performing fine motor skills with their hands. Additionally, it has been reported that IWPD more often face different barriers regarding social and/or political communication and accessibility, among others, which affect their general well-being and prevent them from having equal opportunities [5]. Severe architectural, economic, and educational barriers hinder their functioning. In addition, social barriers resulting from indifferent or negative attitudes play a key role in developing their self-awareness and motivational sphere in the professional, social, and cultural space. Social perception of IWPD influences their personal choices, decisions, mindset, awareness, and separateness in the form of “us” and “them.” Negative social attitudes create prejudices, fears, and negative patterns of behavior [6].

Several disability support plans must be implemented to allow IWPD to maintain their independence and live successfully in the community. Some IWPD may want guidance from their general practitioner or a specialist, while others may require a multidisciplinary team of medical professionals to oversee different areas of their treatment, including physiotherapists, occupational therapists, and speech therapists [2]. However, despite varying degrees of disability, epidemiologic studies on disability have emphasized the importance of regular physical activity (PA) in improving the health of IWPD [7].

Relevant studies have proven that PA has a twofold impact—enhancing physical fitness and improving physical and mental well-being—enabling IWPD to enjoy its benefits, boosting their self-confidence, and potentially reducing their feelings of inferiority [8,9,10]. The Centers for Disease Control and Prevention [11] stated that PA is crucial for preserving health, well-being, and quality of life. It can aid in weight management; enhance mental well-being; and reduce the risk of premature mortality, heart disease, type 2 diabetes, and some malignancies. Engaging in PA can help IWPD achieve increased societal integration [12]. Previous studies have also observed that PA has psychological benefits for IWPD, improving self-esteem, autonomy, goal achievement, personal development, self-control, and self-confidence [10]. Furthermore, it has been shown that PA has social benefits because it favors inclusion and social relations [3].

Engaging in any form of PA that elevates the heart rate might enhance overall health. Any activity is preferable to none. The World Health Organization (WHO) guidelines recommend that children and adolescents (ages 5 to 17) with disabilities engage in at least 60 min per day of moderate- to high-intensity PA, primarily aerobics, throughout the week. At least three days per week, vigorous-intensity aerobic activities, as well as muscle- and bone-strengthening exercises, should be practiced. Adults (aged 18 years and over) living with disabilities, on the other hand, should engage in 150–300 min of moderate-intensity aerobic PA, 75–150 min of vigorous-intensity aerobic PA, or an equivalent combination of moderate- and vigorous-intensity PA weekly. They should also perform muscle-strengthening activities involving all major muscle groups at moderate or higher intensity at least two days a week, as they provide additional health benefits. The WHO guidelines also recommend that disabled older people engage in varied, multicomponent PA at least three times per week. This activity should focus on functional balance and strength training at a moderate to high intensity with the goal of improving functional capacity and reducing the risk of falls. To optimize their health advantages, adults with disabilities can increase moderate-intensity aerobic PA to more than 300 min, do more than 150 min of vigorous-intensity aerobic PA, or do an equivalent combination of moderate- and vigorous-intensity activity throughout the week [13].

However, research indicates that IWPD engage in less PA than those without disabilities, leading to a high incidence of sedentary behavior. Ginis et al. [14] indicated that individuals with various disabilities are 16–62% less likely to meet prescribed PA levels and are at a higher risk of developing health issues due to a lack of PA. In a study of Spanish adults with disabilities, Ramírez et al. [15] found that only 29% of participants met the WHO’s daily recommendation of 60 min of PA, with 51% of women and 40.7% of men classified as sedentary.

Based on a study of research articles from 1980 to 2009, Saebu [16] found that IWPD are often less physically active than the general population. He claimed that PA levels in IWPD varied according to their types and degrees of functioning and impairment, showing a positive correlation between decreased functioning and reduced PA. This correlation was most apparent in populations with significant activity limits, such as those with MS, CP, and spinal cord injury. These findings support the assertion that there is a connection between general and diverse disability groups and increased inactivity and that having any impairment decreases mean activity levels [17]. More recently, Bloemen et al. [18] and Sit et al. [19] discovered that youths with physical disabilities have high levels of physical inactivity in their daily lives. Individuals with conditions such as CP are notably less physically active than their peers without these conditions [20]. However, a systematic review by Seemüller et al. [21] found that PA intensity impacted PA duration in children and adolescents who primarily use a wheelchair for mobility. Bloemen et al. [22] found a mean of 94 min of moderate to vigorous PA per day, Sol et al. [23] found a mean of 98 min of PA across all intensities per day, and Bloemen et al. [18] found a mean of 72 min of habitual PA per day, meeting the WHO-recommended level.

Disability is a substantial social and economic issue in Saudi Arabia. According to the General Authority for Statistics [24], 7.1% (n = 1,445,723) of individuals living in Saudi Arabia have disabilities, comprising 52.2% males and 47.8% females, with most having mobility or physical disabilities (n = 833,136, 2.53%; [24]). These rates are expected to increase due to continued increases in health risk factors such as obesity, physical inactivity, traffic accidents, and chronic diseases. The increasing number of IWPD is a constant challenge for the government and healthcare stakeholders in Saudi Arabia, requiring a comprehensive health approach to reduce risk factors that is based on outcomes that reflect the reality of IWPD in the country.

Given the importance of PA in improving physical, psychological, and social well-being, it is vital to assess its prevalence among Saudis with physical disabilities and identify the factors affecting its promotion among this population. Previous studies in Saudi Arabia have examined the prevalence of PA in the general population [25,26,27,28,29]. A recent nationwide survey reported that 82.6% of adults in Saudi Arabia were physically inactive [26].

Few studies have evaluated PA levels among IWPD in Saudi Arabia; only two conducted by Zahra et al. in 2022 have explored this topic. The first study examined the disparity in PA engagement and sedentary time between individuals with and without disabilities and how these factors relate to psychological quality of life [7]. The second study determined the PA levels of individuals with and without physical disabilities in Saudi Arabia, evaluating their perception of environmental quality of life and its influence on PA [30]. Therefore, the present study aimed to evaluate the prevalence, pattern, and levels of PA among IWPD in Saudi Arabia using the Arabic version of the Physical Activity Scale for Individuals with Physical Disabilities (PASIPD-AR). We also explored the association between PA and the type of disability, mobility assistive devices, and demographic characteristics of IWPD.

This study investigated the following research inquiries:

1. What is the extent of weekly PA among IWPD in Saudi Arabia?
2. What type of PA is most appealing to IWPD in Saudi Arabia?
3. Are there associations between PA and the type of disability, mobility assistive devices, and demographic characteristics of IWPD?

## 2. Materials and Methods

### 2.1. Data Collection and Participants

This cross-sectional study used an online survey and was conducted between 1 November 2023 and 31 January 2024. The contact information for 300 IWPD was collected from three social rehabilitation centers in the Eastern Province of Saudi Arabia, including their telephone number, cell number, email address, and WhatsApp number. They received an invitation to participate, accompanied by a concise explanation of the study protocol and inclusion criteria. The conditions indicated that participants must be at least 18 years old, have a verified physical disability, and be able to read and write. Once their participation confirmations were received, a digital copy of the survey was provided by email or WhatsApp. The participants were invited to follow the links in the email and sign an informed consent form on the first page. A total of 242 IWPD consented to participate in our study and acknowledged and agreed to the terms outlined above before proceeding with the survey. After clicking “I Agree,” the participant was sent a two-part online survey hosted on Google Forms, which was predicted to take 10 min to complete. The results (N = 242) were downloaded and confirmed for accuracy. Incomplete questionnaires or questionnaires containing incorrect answers were excluded from the analysis (n = 4). A total of 238 respondents were included in the study sample, comprising 148 males and 90 females, resulting in a completion percentage of 80.7%. This study was approved by the Research Ethics Committee of King Faisal University, Al-Ahsa, Saudi Arabia (reference number: KFU-REC-2023-JUN-ETHICS1091).

### 2.2. Instrumentation

Data were collected using a two-part questionnaire with 27 items. The first part consisted of 14 items that collected information about demographics, body composition, self-rated health, self-rated fitness, type of disability, and use of mobility assistive devices. Self-rated health and fitness were evaluated using a three-point Likert scale with bad, good, and outstanding categories. The second part was the PASIPD-AR, a scale designed to assess PA levels in IWPD. Initially developed in English, the PASIPD was later translated and adapted to the Saudi context by Alhumaid et al. [31]. The straightforward structure of the scale makes it ideal for use in survey-based research involving many participants. Additionally, the PASIPD can distinguish between individuals with good health and those with bad health, as well as between participants of different ages, levels of physical activity (moderate, high, or low), and whether they are receiving auxiliary care [32]. Similar to other well-established self-report physical activity measures utilized in the general population [33] and in populations suffering from chronic neurological diseases, such as brain injury [34], the PASIPD has shown test–retest reliability and criterion validity [35]. The PASIPD has been validated for use in individuals who have a range of physical disabilities, and it also tackles the challenges associated with measuring PA in this population [36].

### 2.3. PASIPD-AR

The PASIPD-AR is an Arabic adaptation of Washburn et al.’s [32] Physical Activity Scale for Individuals with Physical Disabilities. It consists of 13 items documenting the respondent’s activity and inactivity patterns (sedentary, leisure, domestic, and occupational behaviors) in the previous week, including the number of days and hours spent on each activity. The PASIPD-AR includes four latent factors instead of the five in the original English version. Factor 1 covers home repair, lawn mowing, and gardening activities (HRA; items 9, 10, and 11). Factor 2 covers household activities (HHA; items 7, 8, and 12). Factor 3 covers light to vigorous sports and recreational activities (SRA; items 3, 4, 5, and 6). Factor 4 covers occupational and transportation activities (OTA; items 2 and 13). The respondent is required to remember and report the frequency of engaging in activities during the past seven days as never/seldom (1–2 days/week), occasionally (3–4 days/week), or often (5–7 days/week), as well as the mean daily duration of participation (<1, 1–2, 2–4, and >4 h). The hours per day for the occupational item are categorized as <1, 1–4, 5–8, and ≥8 h. The PASIPD-AR score is calculated by multiplying the mean daily duration of each activity by its respective metabolic equivalent (MET) value. The PASIPD-AR scores range from 0.0 MET h/day (no activities completed) to 199.5 MET h/day (the highest duration of days and hours for all activities undertaken).

### 2.4. Statistical Analysis

To fulfill the initial two research objectives, we computed the mean and standard deviation of PASIPD-AR scores and those for the different forms of PA in which participants were involved. In addition, van Remoortel et al. [37] suggested that a non-bout target of 80 min/day using a 3 MET moderate to vigorous PA threshold was equivalent to 30 min/day of moderate to vigorous PA commonly used to evaluate compliance with the minimum standards set by the WHO [13]. Therefore, the participants were classified into two distinct cohorts: those who failed to meet the minimum criteria and those who met the necessary standards. The prevalence of PA was compared by disability type, mobility assistive device, and demographic variables using a chi-square test.

Due to the non-normal distribution and positive skewness of the PASIPD-AR scores and components, the data were logarithmically transformed. Additionally, the final research objective was examined using multivariate analysis of covariance with age (in years) as a covariate. Effect sizes were calculated as partial eta squared (*η^2^*) and interpreted as follows: *η*^2^ = 0.01 indicates a small effect, *η*^2^ = 0.06 indicates a medium effect, and *η*^2^ = 0.14 indicates a large effect. The requisite assumptions were assessed by targeted testing using SPSS software (version 26; IBM, Armonk, NY, USA), and the significance level was set at *p* < 0.05.

## 3. Results

The sample of 238 participants was predominantly male (62.2%). Among participants, 19.3% had a SD, 14.7% had PMD, 15.1% had MS, 17.6% had CP, 16.4% had poliomyelitis, and 16.8% had a leg or foot amputation (LFA). The participants’ mean age was 39.76 ± 12.19 years, with 32.8% aged 24–34, 27.7% aged 35–44, 29% aged 45–54, and 10.5% aged 55–64. Their mean body mass index was 28.17 ± 8.61 kg/m2, their height was 159.25 ± 14.22 cm, and their weight was 71.22 ± 22.99 kg. Additional characteristics stratified by gender and type of disability were incorporated into Table 1.

### 3.1. Prevalence of PA

**Figure 1:**
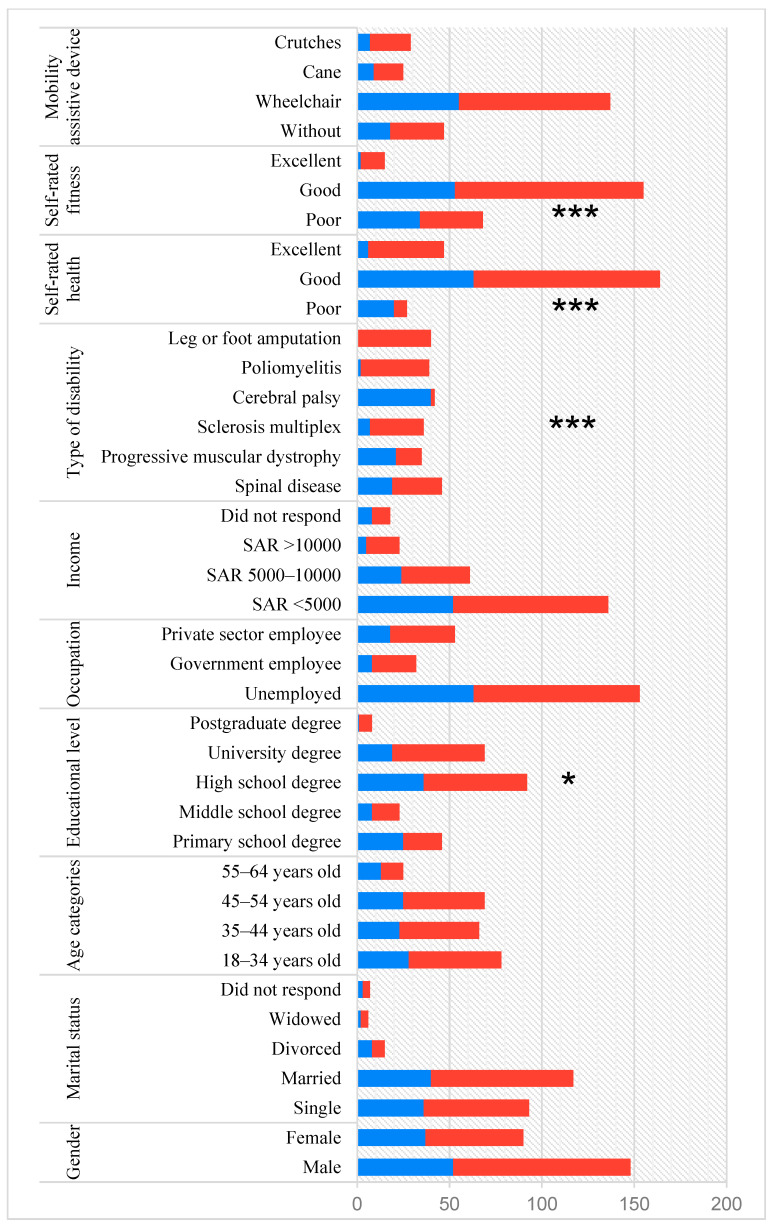
The prevalence of PA among IWPD in Saudi Arabia. Inactive participants are shown in blue and active participants in red. Key: *, *p* < 0.05; ***, *p* < 0.001.

The PASIPD-AR scores revealed that 62.6% of the participants (64.9% of males and 58.9% of females) met the minimum standards specified by the WHO, compared with 37.4% who reported an insufficient level of participation, including 20 participants (8.4%) who indicated that they did not engage in any form of physical activity. PA prevalence did not differ significantly between males and females (χ^2^ = 0.854, *p* = 0.355). However, it did differ significantly by educational level (χ^2^ = 10.82, *p* = 0.029), type of disability (χ^2^ = 114.16, *p* < 0.001), self-rated health (χ^2^ = 27.77, *p* < 0.001), and self-rated fitness (χ^2^ = 9.01, *p* = 0.011). In contrast, PA prevalence did not differ significantly by sex, age category, marital status, occupation, mean family income, or the use of mobility assistive devices (Figure 1).

### 3.2. Level of PA and Influencing Factors

#### 3.2.1. Overall PA

The participants’ mean PASIPD-AR score was 10.33 ± 10.67 MET h/day out of a maximum possible score of 199.5 MET h/day, ranging from 0 to 49.28 MET h/day, indicating that PA among IWPD in Saudi Arabia is primarily low. After adjusting for age, PASIPD-AR scores did not differ significantly by sex, occupation, mean family income, or self-rated fitness (Table 2). Nevertheless, they did differ significantly by the type of disability (*η*^2^ = 0.436, *p* < 0.001) and use of mobility assistive devices (*η*^2^ = 0.082, *p* < 0.001) and, to a lesser extent, by marital status (*η*^2^ = 0.052, *p* = 0.023), educational level (*η*^2^ = 0.053, *p* = 0.022), and self-rated health (*η*^2^ = 0.045, *p* = 0.008).

Specifically, participants who were divorced exhibited the highest levels of PA, differing significantly from those who chose not to disclose their marital status (*p* = 0.015). Among types of disabilities, those with CP were found to be the least physically active (all *p* < 0.001), while those with poliomyelitis were the most physically active, differing significantly from those with SD (*p* = 0.018) or an LFA (*p* < 0.001). Furthermore, those diagnosed with SD exhibited lower levels of PA than those with MS (*p* = 0.009). Conversely, those with PMD were more physically active than those with an LFA (*p* = 0.048). Additionally, those with MS were more physically active than those with an LFA (*p* < 0.001). Ultimately, those who relied on canes for mobility were less physically active than those who did not require mobility assistive devices (*p* = 0.004). This difference in activity level was even more pronounced for those using wheelchairs (*p* < 0.001) or crutches (*p* = 0.037). 

#### 3.2.2. Home Repair, Lawn Mowing, and Gardening Activities

The participants’ mean PASIPD-AR HRA score was 0.59 ± 1.35 MET h/day, ranging from 0 to 8.56 MET h/day, suggesting that most engaged in these activities at a low level. When adjusting for age, significant differences with moderate effect sizes were observed for marital status (*η*^2^ = 0.069, *p* = 0.005) and type of disability (*η*^2^ = 0.060, *p* = 0.025; Table 3). In addition, significant differences with small effect sizes were observed for occupation (*η*^2^ = 0.040, *p* = 0.014), mean family income (*η*^2^ = 0.038, *p* = 0.041), and mobility assistive devices (*η*^2^ = 0.040, *p* = 0.035). The pairwise comparisons revealed that unmarried participants performed HRA less than married participants (*p* = 0.040) but more than those who chose not to disclose their marital status (*p* = 0.004). Divorced participants performed HRA more than those who were married (*p* = 0.009) or declined to respond (*p* = 0.001). Unemployed participants performed HRA less than those who worked for the government (*p* = 0.031) or private sector (*p* = 0.008). Participants who earned SAR 5000–10,000 performed HRA less than those who earned more than SAR 10,000 (*p* = 0.013). Participants with MS performed HRA more than those with PMD (*p* = 0.013), CP (*p* = 0.002), and LFA (*p* = 0.003) but not SD (*p* = 0.145). However, they performed HRA less than those with poliomyelitis (*p* = 0.013). Participants who used a wheelchair as a mobility assistive device performed HRA less than those who used a cane (*p* = 0.028) or crutches (*p* = 0.045). Age did not appear to have any significant impact on the outcomes (*p* >0.05).

#### 3.2.3. Household Activities

The participants’ mean PASIPD-AR HHA score was 2.12 ± 3.34 MET h/day, ranging from 0 to 18.88 MET h/day, suggesting that they were mostly engaged in these activities at a modest level. Regardless of the age of the participants, the scores differed significantly by sex (*η*^2^ = 0.029, *p* = 0.013) and the type of disability (*η*^2^ = 0.126, *p* < 0.001; Table 3). The pairwise comparisons revealed that participants with poliomyelitis performed HHA significantly more than those with SD (*p* < 0.001), PMD (*p* = 0.046), MS (*p* = 0.016), CP (*p* < 0.001), or LFA (*p* < 0.001). Furthermore, those with PMD (*p* = 0.006) or MS (*p* = 0.032) performed HHA more than those with an LFA. Participants with PMD performed HHA significantly more than those with SD (*p* = 0.022).

#### 3.2.4. Light to Vigorous Sport and Recreational Activity

The participants’ mean PASIPD-AR SRA score was 2.33 ± 4.17 MET h/day, ranging from 0 to 20.73 MET h/day, indicating that most engaged in SRA activities at a low level. The scores differed significantly by the type of disability (*η*^2^ = 0.060, *p* = 0.006) and education level (*η*^2^ = 0.088, *p* = 0.001), regardless of age (Table 4). Additionally, significant differences with minimal effect sizes were observed for self-rated health (*η*^2^ = 0.280, *p* = 0.050), occupation (*η*^2^ = 0.035, *p* = 0.023), and type of disability (*η*^2^ = 0.052, *p* = 0.046). The pairwise comparisons revealed that participants with a primary school educational level performed SRA less than those with a university degree (*p* = 0.005), postgraduate degree (*p* = 0.007), or middle school educational level (*p* = 0.016). In addition, individuals with a high school educational level had significantly lower levels of engagement in SRA compared to those with a university degree (*p* = 0.003) or postgraduate degree (*p* = 0.011). Moreover, unemployed participants performed SRA significantly less than those who worked for the government (*p* = 0.026) and the private sector (*p* = 0.026). Furthermore, participants with MS performed SRA significantly more than those with PMD (*p* = 0.017), CP (*p* = 0.028), or SD (*p* = 0.001). In addition, participants who reported their health state as poor performed SRA less than those who reported their health as good (*p* = 0.019) or outstanding (*p* = 0.030). Finally, engagement in SRA differed significantly between participants who used a cane for mobility assistance and those who used a wheelchair (*p* = 0.003) or crutches (*p* = 0.001).

#### 3.2.5. Occupational and Transportation Activities

The participants’ mean PASIPD-AR OTA score was 5.29 ± 7.52 MET h/day, ranging from 0 to 30.01 MET h/day. Regardless of participants’ age, the type of disability had the greatest impact on engagement in OTA activities (*η*^2^ = 0.216, *p* < 0.001). In addition, engagement in OTA differed significantly with moderate effect sizes by marital status (*η*^2^ = 0.061, *p* = 0.010), use of mobility assistive devices (*η*^2^ = 0.096, *p* < 0.001), and occupation (*η*^2^ = 0.075, *p* < 0.001). Moreover, engagement in OTA differed significantly with small effect sizes by educational level (*η*^2^ = 0.044, *p* = 0.051) and self-rated health (*η*^2^ = 0.054, *p* = 0.003; Table 4). The pairwise comparisons revealed that unmarried participants engaged in OTA less than divorced participants (*p* = 0.015). In addition, those who chose not to disclose their marital status engaged in OTA less than those who were married (*p* = 0.007), divorced (*p* = 0.001), or widowed (*p* = 0.028). Participants with a middle school educational level performed OTA more than those with a primary school educational level (*p* = 0.013) or university degree (*p* = 0.009). Unemployed participants performed OTA significantly less than those employed by the government (*p* < 0.001) or the private sector (*p* = 0.004). Participants with CP performed OTA significantly less than those with an LFA (*p* = 0.006) and other disabilities (*p* < 0.001). In addition, engagement in OTA differed significantly between participants with an LFA and those with PMD (*p* = 0.003), MS (*p* = 0.001), or poliomyelitis (*p* = 0.001). Furthermore, participants who rated their health as bad performed less OTA than those who rated their health as excellent (*p* = 0.001). Finally, participants who used a wheelchair for mobility performed OTA significantly more than those who used a cane (*p* = 0.001), used crutches (*p* = 0.005), or did not use any mobility aids (*p* = 0.001).

## 4. Discussion

This study aimed to assess the prevalence, distribution, and level of PA among IWPD in Saudi Arabia using a specialized questionnaire, the PASIPD-AR. It also examined the impact of demographic characteristics, type of disability, and use of mobility assistive aids on PA levels. The findings showed that 62.6% of the participants (64.9% of males and 58.9% of females) met the minimum guidelines set by the WHO, with 8.4% reporting no participation in PA whatsoever. The participants’ mean PASIPD-AR score was 10.33 ± 10.67 MET h/day, with a maximum possible score of 199.5 MET h/day, ranging from 0 to 49.28 MET h/day.

Using the Arabic version of the International Physical Activity Questionnaire Short Form (IPAQ-SF), Zahra et al. [7] noted that only 46% of their 359 participants (67.7% without disability and 32.3% with disability) met the minimum level of PA, including 49.1% of those with disability and 44% of those without disability. The divergence between our results and those of Zahra et al. [7] can be attributed to methodological differences. The PASIPD-AR evaluates the level of an active lifestyle, while the IPAQ-SF only evaluates activities conducted in bouts lasting more than 10 min. In addition, while Zahra et al. [7] classified the individuals based on a cutoff of 600 MET min/week, we established a minimum threshold of 240 MET min/day [37].

Compared to the general population, our study found that PA levels among the participants were higher than reported in the WHO National Diabetes Profile 2016 in Saudi Arabia. According to that profile, 41.5% of adults in Saudi Arabia were physically active, including 47.9% of men and 32.3% of women [38]. Our findings also differed from those of Al-Zalabani et al. [39], who reported a 43.4% prevalence of PA in the total Saudi population (39.9% in men and 27.1% in women). Al-Zalabani et al. [39] observed that 16.8% of the population participated in moderate PA, whereas 16.6% engaged in high PA. In contrast, Alqahtani et al. [26] showed that, of 26,000 families from 13 administrative regions in Saudi Arabia, only 17.40% of adults aged ≥15 years engaged in PA for at least 150 min per week, with the remaining 82.60% not participating in any PA.

Heath and Levine [40] reported that 20.6–50.0% of adults with disabilities met the WHO guidelines for PA in high-income countries, compared to 23.4–50.0% in low- and middle-income countries. The reported prevalence of PA among individuals without disabilities in high- and low-income nations was estimated to be 50% to 80% [41,42,43]. In a study by Ellis et al. [44], 223 individuals with a mean age of 45.4 ± 10.8 years completed a web-based survey. Their mean total PA score was 20.5 ± 16.8 MET h/day, which equates to around five hours per week of vigorous walking or quick wheelchair movement, according to the IPAQ Research Committee [45].

Our findings also demonstrated that, regardless of age, PASIPD-AR scores differed significantly by the type of disability. Participants with poliomyelitis exhibited the highest levels of PA, indicating that they engaged in more PA than those with other conditions, including SD, CP, and LFA, both on a daily and weekly basis. This behavior was primarily noticed in the context of home repair, household, occupation, and transportation activities. Ganesh et al. [46] observed that 96 university students in India with polio had a mean MET score of 27.10 h per day. This cross-sectional study also revealed that individuals with polio were primarily engaged in domestic tasks, spending a mean of around three hours per day on such activities [46]. Nonetheless, Winberg et al. [47] suggested that restrictions on the PA of individuals experiencing the late effects of polio cannot be fully explained by parameters such as knee muscular strength and gait performance alone. They argued that other aspects must be investigated to better understand their role.

Conversely, our participants with LFA exhibited the lowest level of PA, particularly in household, occupation, and transportation activities. Van Helm et al. [48] reported a similar observation, confirming that lower limb amputation adversely affects physical ability and increases discomfort. Davie-Smith et al. [49] also observed that the ability to walk with a prosthesis became more significant for individuals with lower limb amputations because it helped them live independently and enhanced their engagement in social activities. However, the ability to walk is affected by several factors, such as the extent of amputation, other medical conditions, psychological drive, living conditions, and social capabilities [49]. Individuals with a lower limb amputation experience altered energy expenditure during walking. According to van Schaik et al. [50], walking with a prosthesis demands higher oxygen consumption than walking without physical impairments. Moreover, oxygen consumption was higher with amputations closer to the body and when the walking speed was higher, which might adversely affect PA patterns [48].

Unfortunately, no previous studies have examined PA levels among amputees in Arab and Islamic societies. Abouammoh et al. [51] argued that culture significantly influences an individual’s lifestyle, views, and attitudes, as well as their family and social networks. AlSofyani et al. [52] reported that a significant majority of amputees in Saudi Arabia, almost two-thirds, do not avail themselves of rehabilitation programs for undisclosed reasons. The economic ramifications of providing medical care to those who have undergone amputation are substantial, and neglecting to address their requirements can worsen their outcomes. Abouammoh et al. [51] reported that local cultural and social factors can contribute to the sense of disability experienced by amputees. Individuals construct their sense of self and perception of their physical appearance based on the perspectives of others. Given this perspective, Saudi amputees prefer not to receive compassion or assistance from others when they face logistical challenges.

Our study also observed that using a mobility assistive device significantly affected PA levels. Participants who relied on canes for mobility were less physically active than those who did not use mobility assistive devices. This difference in PA was even more pronounced for those who used wheelchairs or crutches. De Hollander and Proper [53] observed that adults with physical disabilities engaged in less PA (−37.7%) than those without physical or sensory impairments. Among assistive devices, the greatest differences were observed among IWPD who used mobility aids (−49.8%), such as transport chairs and recliners. Moreover, despite additional adjustments for self-reported motor limitations, these disparities remained significant: −21.9% and −29.0%, respectively. Carver et al. [54] asserted that while the primary objective of assistive mobility devices is to enhance quality of life, they may also be regarded as detrimental to an individual’s existence. Inadequately adapted devices may adversely affect physical functioning, quality of life, and occupational activity [55]. Jutai and Day [56] found that the owners of these devices occasionally neglect or fail to use them. Conversely, Kaye et al. [57] found that wheelchair users had the lowest employment level and the greatest activity and functional limitations. A potential correlation exists between economic and social oppression and functional and activity limitations. Individuals who lack access to technology face constraints in their pursuit of education, employment, and leisure activities [58].

Our results revealed that marital status primarily has moderate yet significant effects on HRA and OTA levels, educational attainment on SRA levels, and occupation on OTA levels. Our findings regarding the effects of marital status on PA were inconclusive. Some studies have indicated that married adults participate in higher levels of PA [59,60], while others have indicated that they engage in lower levels of PA [61]. This inconclusive evidence indicates that the effects of marriage on health and health behaviors may vary across married couples. The levels of marital support, such as feeling loved, cared for, and listened to, as well as marital strain, such as feeling bothered, upset, and experiencing conflicts, were associated with increased PA [62]. Nomaguchi and Bianchi [61] found that married men engage in 2 h and 50 min less physical exercise every two weeks than unmarried men. The financial and familial obligations associated with marriage may also account for disparities in PA levels between married and single males. Married individuals exhibited a high level of engagement in their children’s education [63]. The prioritization of their responsibilities as providers, dads, spouses, and community members hindered their engagement in PA, becoming a significant obstacle to increasing their participation in such activities.

Moreover, individuals with disabilities often have financial constraints, low rates of employment, and precarious job situations. Consequently, they must allocate significant funds toward training and rehabilitation therapies. In addition, most spouses with disabilities typically have poor incomes, which reduces the overall economic resilience of their family. Therefore, those with disabilities who desire to begin a family must first secure employment. Alternatively, if they cannot generate revenue, they will be unable to support their family financially and ensure a basic standard of living after marriage. Moreover, those with disabilities are burdened with elevated care expenses. A marriage between unemployed individuals will inevitably escalate both their loads and be unfavorable to a dynamic way of living. Individuals with disabilities can benefit from pursuing higher education because it can enhance their chances of securing employment and enable them to engage in consistent PA. Individuals who pursue education will likely experience several advantages, such as personal independence, community integration, and employment, besides other social, physical, and psychological benefits. A higher education provides individuals with numerous advantages that can encourage PA, such as increased awareness of its benefits, a stronger sense of personal control and self-efficacy for PA, healthier influences from social network members, and improved access to resources that support PA [64,65].

This study had some limitations. Firstly, there was a significant disparity in the number of females compared to male participants, which could be perceived as a disadvantage. This issue may have influenced the correlation between sex and PA levels. Secondly, we did not collect further data regarding the participants’ disability, such as the length, severity, or consequences of prior diseases. Thirdly, it is unclear from the available information whether the participants required personal assistance for other vocational activities and if they were obligated to switch from one assistive device to another. Fourthly, insufficient attention was paid to other concerns related to physical constraints that affect PA, such as family involvement, psychological traits, PA promoters, and PA barriers. Consolidating all motor disabilities into a single category also failed to allow for the specific requirements of each patient to be identified. Future research that evaluates the precise type of motor handicap and its corresponding outcomes will generate more substantial interest. Fifthly, the values recorded and analyzed were solely based on self-reported responses to a questionnaire. As a result, the likelihood of recollection bias and social desirability results cannot be excluded. Recall bias occurs when contributors forget specific occurrences, quantities, or frequencies [66]. However, asking participants about typical or common activities, as well as tracking the amount of gardening, house maintenance, or sports or video game sessions in the previous seven days, may have lowered the possibility of recall bias. Finally, the questionnaire was employed as an indirect means of determining PA levels. The use of direct measurement methods, such as actimetry, can provide significantly higher precision.

## 5. Conclusions

According to the PASIPD-AR score, 62.6% of the participants (64.9% of males and 58.9% of females) met the minimum guidelines established by the WHO, whereas 37.4% reported an insufficient level of participation, including 20 participants (8.4%) who indicated that they did not engage in any form of physical activity. After adjusting for age, PA levels differed significantly by the type of disability and use of mobility assistive devices. More specifically, significant differences in HRA with modest effect sizes were observed for marital status and the type of disability. In addition, HHA levels differed significantly by the type of disability. Participants affected by poliomyelitis engaged in HHA more than those with other conditions, such as SD, PMD, MS, CP, and LFA. The SRA levels were influenced by the type of disability and educational level. Participants with a primary school educational level had lower SRA levels than those with a university degree, postgraduate degree, or middle school educational level. SRA levels were significantly lower among unemployed participants than among government and private sector employees. Finally, the type of disability, marital status, use of mobility assistive devices, and occupation were found to affect OTA levels significantly but moderately.

## Figures and Tables

**Table 1:** Age and anthropometric characteristics of IWPD in Saudi Arabia, stratified by gender and type of disability.

			Age (Years)		Weight (kg)		Height (cm)		BMI (kg/m^2^)	
Gender	Type of disability	N	Mean	SD	Mean	SD	Mean	SD	Mean	SD
Male	Spinal disease	35	39.31	13.83	73.46	18.96	163.83	14.59	27.50	7.12
	Progressive muscular dystrophy	25	38.36	10.93	82.00	33.24	170.36	7.57	28.16	10.62
	Multiple sclerosis	15	32.93	10.28	69.87	22.44	166.33	6.37	25.41	9.03
	Cerebral palsy	22	42.45	13.23	67.73	19.35	160.14	8.19	26.61	8.61
	Poliomyelitis	24	48.38	7.59	78.71	21.67	157.46	10.06	31.55	7.01
	Leg or foot amputation	27	36.11	11.35	66.93	24.74	159.74	20.52	26.08	8.52
	Total	148	39.86	12.30	73.34	24.03	162.86	13.45	27.67	8.53
Female	Spinal disease	11	39.64	9.57	61.36	13.57	152.55	13.93	26.40	4.54
	Progressive muscular dystrophy	10	41.20	7.76	69.70	11.62	156.80	6.91	28.26	3.56
	Multiple sclerosis	21	36.76	12.98	63.48	17.21	154.29	9.85	26.36	5.69
	Cerebral palsy	20	38.75	17.06	65.20	22.56	149.75	23.49	30.34	11.93
	Poliomyelitis	15	46.20	3.47	75.80	13.09	151.53	5.87	32.97	4.99
	Leg or foot amputation	13	36.69	11.03	73.00	35.58	157.31	4.79	29.31	13.73
	Total	90	39.61	12.07	67.72	20.82	153.32	13.50	28.99	8.73
Total	Spinal disease	46	39.39	12.84	70.57	18.43	161.13	15.08	27.23	6.57
	Progressive muscular dystrophy	35	39.17	10.10	78.49	29.11	166.49	9.58	28.19	9.11
	Multiple sclerosis	36	35.17	11.92	66.14	19.52	159.31	10.39	25.97	7.17
	Cerebral palsy	42	40.69	15.10	66.52	20.72	155.19	17.82	28.39	10.36
	Poliomyelitis	39	47.54	6.36	77.59	18.69	155.18	9.08	32.10	6.28
	Leg or foot amputation	40	36.30	11.11	68.90	28.39	158.95	17.01	27.13	10.43
	Total	238	39.76	12.19	71.22	22.99	159.25	14.22	28.17	8.61

Note: SD, standard deviation.

**Table 2:** PA levels among IWPD in Saudi Arabia stratified by type of disability, mobility assistive devices, and demographic characteristics.

		*N*	PASIPD-AR Score	*p*/*η*^2^
Sex	Male	148	10.244 ± 10.722	NS
	Female	90	10.475 ± 10.649	
Marital status	Single	93	9.197 ± 9.506	0.023/0.052
	Married	117	10.429 ± 10.536	
	Divorced	15	17.426 ± 18.06	
	Widowed	6	12.367 ± 2.351	
	Did not respond	7	6.826 ± 6.112 ^c^	
Educational level	Primary school	46	7.640 ± 12.187	0.022/0.053
	Middle school	23	12.433 ± 10.834	
	High school	92	9.794 ± 9.688	
	University degree	69	11.717 ± 11.010	
	Postgraduate degree	8	14.003 ± 5.914	
Occupation	Unemployed	153	8.501 ± 9.301	NS
	Government employee	32	16.628 ± 12.891	
	Private sector employee	53	11.814 ± 11.431	
Mean family income	SAR < 5000	136	8.719 ± 9.536	NS
	SAR 5000–10,000	61	11.739 ± 11.009	
	SAR > 10,000	23	13.704 ± 11.846	
	Did not respond	18	13.440 ± 14.245	
Type of disability	Spinal disease	46	11.186 ± 11.056 ^c,d,e^	<0.001/0.436
	Progressive muscular dystrophy	35	12.134 ± 10.398 ^d,f^	
	Multiple sclerosis	36	14.240 ± 6.534 ^d,f^	
	Cerebral palsy	42	1.175 ± 2.901 ^e,f^	
	Poliomyelitis	39	18.912 ± 13.053 ^f^	
	Leg or foot amputation	40	5.503 ± 6.168	
Self-rated health	Poor	27	3.870 ± 7.883	0.008/0.045
	Good	164	9.769 ± 10.522	
	Excellent	47	16.009 ± 10.042	
Self-rated fitness	Poor	68	5.556 ± 7.275	NS
	Good	155	11.865 ± 11.287	
	Excellent	15	16.139 ± 10.027	
Mobility assistive device	Unaided	47	9.277 ± 9.623	<0.001/0.082
	Wheelchair	137	11.482 ± 11.375	
	Cane	25	6.093 ± 8.335 ^a,b,d^	
	Crutches	29	10.261 ± 9.978	

Note: NS, not significant; *η*^2^, partial eta squared; ^a,b,c,d,e,f^, the subgroups in each variable in alphabetical order.

**Table 3:** Levels of home repair, lawn mowing, gardening, and household activities among IWPD in Saudi Arabia stratified by type of disability, mobility assistive devices, and demographic characteristics.

		*N*	Home Repair, Lawn Mowing, and Gardening Activities	*p*/*η*^2^	Household Activities	*p*/*η*^2^
Sex	Male	148	0.609 ± 1.445	NS	1.514 ± 2.533	0.013/0.029
	Female	90	0.549 ± 1.180		3.122 ± 4.168	
Marital status	Single	93	0.539 ± 1.361	0.005/0.069	1.707 ± 3.040	NS
	Married	117	0.541 ± 1.277 ^a^		2.158 ± 3.256	
	Divorced	15	1.051 ± 2.043 ^b^		3.879 ± 4.932	
	Widowed	6	1.453 ± 0.888		1.863 ± 2.100	
	Did not respond	7	0.251 ± 0.235 ^a,c^		3.500 ± 4.389	
Educational level	Primary school	46	0.587 ± 1.603	NS	2.136 ± 3.736	NS
	Middle school	23	1.045 ± 1.910		1.181 ± 1.783	
	High school	92	0.444 ± 0.917		2.225 ± 3.685	
	University degree	69	0.678 ± 1.482		2.111 ± 2.856	
	Postgraduate degree	8	0.110 ± 0.204		3.659 ± 3.882	
Occupation	Unemployed	153	0.456 ± 1.221	0.014/0.040	1.857 ± 3.190	NS
	Government employee	32	0.916 ± 1.193 ^a^		2.458 ± 3.641	
	Private sector employee	53	0.765 ± 1.715 ^a^		2.686 ± 3.523	
Mean family income	SAR <5000	136	0.562 ± 1.432	0.041/0.038	1.971 ± 3.067	NS
	SAR 5000–10,000	61	0.450 ± 0.864		2.108 ± 3.274	
	SAR > 10,000	23	0.821 ± 1.264 ^b^		1.426 ± 2.101	
	Did not respond	18	0.936 ± 2.024		4.198 ± 5.594	
Type of disability	Spinal disease	46	0.685 ± 1.437	0.025/0.060	1.485 ± 2.245 ^e^	<0.001/0.129
	Progressive muscular dystrophy	35	0.272 ± 0.823 ^c^		2.690 ± 4.010 ^a,e,f^	
	Multiple sclerosis	36	1.082 ± 1.733		2.934 ± 3.149 ^e,f^	
	Cerebral palsy	42	0.000 ^c^		0.567 ± 2.146 ^e^	
	Poliomyelitis	39	1.171 ± 1.721 ^c^		4.502 ± 4.594	
	Leg or foot amputation	40	0.348 ± 1.187 ^c^		0.940 ± 1.528 ^e^	
Self-rated health	Poor	27	0.000	NS	0.306 ± 0.644	NS
	Good	164	0.599 ± 1.335		1.884 ± 2.917	
	Excellent	47	0.880 ± 1.665		3.997 ± 4.599	
Self-rated fitness	Poor	68	0.418 ± 1.006	NS	1.233 ± 2.512	NS
	Good	155	0.592 ± 1.402		2.455 ± 3.637	
	Excellent	15	1.299 ± 1.910		2.713 ± 2.652	
Mobility assistive devices	Unaided	47	0.889 ± 1.739	0.035/0.040	2.530 ± 3.588	NS
	Wheelchair	137	0.397 ± 0.904		2.042 ± 3.247	
	Cane	25	0.702 ± 1.750 ^b^		1.626 ± 2.623	
	Crutches	29	0.892 ± 1.844 ^b^		2.267 ± 3.908	

Note: NS, not significant; *η*^2^, partial eta squared; ^a,b,c,e,f^, the subgroups in each variable in alphabetical order.

**Table 4:** Levels of high to vigorous sports and recreational activities and occupational and transport activities among IWPD in Saudi Arabia stratified by disability type, mobility aids, and demographic characteristics.

		*N*	High to Vigorous Sports and Recreational Activities	*p*/*η*^2^	Occupational and Transportation Activities	*p*/*η*^2^
Sex	Male	148	2.529 ± 4.161	0.003/0.042	5.592 ± 8.217	NS
	Female	90	2.007 ± 4.192 ^b^		4.798 ± 6.217	
Marital status	Single	93	2.953 ± 4.353	NS	3.998 ± 5.855	0.010/0.061
	Married	117	1.804 ± 3.570		5.927 ± 8.650 ^e^	
	Divorced	15	3.577 ± 7.280		8.919 ± 8.141 ^a,e^	
	Widowed	6	1.803 ± 2.794		7.247 ± 4.775 ^e^	
	Did not respond	7	0.666 ± 1.137		2.409 ± 2.687	
Educational level (degree)	Primary school	46	1.645 ± 4.716	0.001/0.088	3.271 ± 5.356 ^b^	0.051/0.044
	Middle school	23	4.023 ± 5.486 ^a^		6.184 ± 7.842	
	High school	92	1.306 ± 2.765		5.819 ± 7.461	
	University degree	69	3.330 ± 4.505 ^a,c^		5.598 ± 8.560 ^b^	
	Postgraduate degree	8	4.589 ± 3.608 ^a,c^		5.645 ± 8.200	
Occupation	Unemployed	153	2.770 ± 4.634	0.023/0.035	3.419 ± 4.478	<0.001/0.075
	Government employee	32	1.383 ± 3.045 ^a^		11.872 ± 11.462 ^a^	
	Private sector employee	53	1.636 ± 3.065 ^a^		6.727 ± 9.039 ^a^	
Mean family income	SAR < 5000	136	2.383 ± 4.101	NS	3.803 ± 5.076	NS
	SAR 5000–10,000	61	1.747 ± 3.937		7.434 ± 9.363	
	SAR > 10,000	23	3.401 ± 4.396		8.057 ± 10.900	
	Did not respond	18	2.553 ± 5.144		5.753 ± 8.702	
Type of disability	Spinal disease	46	3.173 ± 4.917 ^c^	0.046/0.052	5.843 ± 7.569 ^d^	<0.001/0.216
	Progressive muscular dystrophy	35	1.273 ± 2.263 ^c^		7.900 ± 9.039 ^d,f^	
	Multiple sclerosis	36	4.696 ± 5.236		5.528 ± 4.889 ^d,f^	
	Cerebral palsy	42	0.197 ± 0.890 ^c^		0.411 ± 0.727	
	Poliomyelitis	39	2.742 ± 5.395		10.497 ± 10.341 ^d,f^	
	Leg or foot amputation	40	2.002 ± 2.720		2.213 ± 3.332 ^d^	
Self-rated health	Poor	27	0.068 ± 0.207	0.050/0.028	3.496 ± 7.873	0.003/0.054
	Good	164	2.375 ± 4.358 ^a^		4.911 ± 7.385	
	Excellent	47	3.479 ± 4.238 ^a^		7.653 ± 7.413 ^a^	
Self-rated fitness	Poor	68	0.721 ± 1.715	NS	3.184 ± 5.591	NS
	Good	155	2.801 ± 4.543		6.018 ± 8.248	
	Excellent	15	4.781 ± 5.658		7.347 ± 5.232	
Mobility assistive device	Unaided	47	1.478 ± 3.155	0.006/0.060	4.380 ± 6.452 ^b^	<0.001/0.096
	Wheelchair	137	2.617 ± 4.593 ^c^		6.426 ± 8.157	
	Cane	25	1.377 ± 2.119		2.388 ± 3.956 ^b^	
	Crutches	29	3.186 ± 4.630 ^c^		3.917 ± 7.512 ^b^	

Note: NS, not significant; *η*^2^, partial eta squared; ^a,b,c,d,e,f^, the subgroups in each variable in alphabetical order.

## Data Availability

The data that support the findings of this study are available from the author upon reasonable request.

## References

[1] Mann W.C., Lane J.P. (1995). Assistive Technology for Persons with Disabilities: The Role of Occupational Therapy.

[2] Clute M.A. (2013). Disability: Physical Disabilities. Eastern Washington University.

[3] Ascondo J., Martín-López A., Iturricastillo A., Granados C., Garate I., Romaratezabala E., Martínez-Aldama I., Romero S., Yanci J. (2023). Analysis of the Barriers and Motives for Practicing Physical Activity and Sport for People with a Disability: Differences According to Gender and Type of Disability. Int. J. Environ. Res. Public Health..

[4] Anaut Bravo S., Arza Porras J., Álvarez Urricelqu M.J. (2017). La exclusión social, una problemática estructural entre las personas con discapacidad. Áreas. Rev. Int. Cienc. Soc..

[5] Cabra H.L. (2017). Barreras para la inclusión de las personas con discapacidad, un escenario de derechos humanos. Heurística Rev. Digit. Hist. Educ..

[6] Tomczyszyn D., Pańczuk A., Szepeluk A. (2022). Attitudes of Students of Social Sciences and Humanities towards People with Physical Disabilities (MAS-PL). Int. J. Environ. Res. Public Health.

[7] Zahra A., Hassan S.-U., Hassan M.S., Parveen N., Park J.-H., Iqbal N., Khatoon F., Atteya M.R. (2022). Effect of physical activity and sedentary sitting time on psychological quality of life of people with and without disabilities; A survey from Saudi Arabia. Front. Public Health.

[8] Arbour-Nicitopoulos K.P., Grassmann V., Orr K., McPherson A.C., Faulkner G.E., Wright F.V. (2018). A Scoping Review of Inclusive Out-of-School Time Physical Activity Programs for Children and Youth with Physical Disabilities. Adapt. Phys. Act. Q..

[9] Martin J.J. (2013). Benefits and barriers to physical activity for individuals with disabilities: A social-relational model of disability perspective. Disabil. Rehabil..

[10] Yang T., Xiao H., Fan X., Zeng W. (2023). Exploring the effects of physical exercise on inferiority feeling in children and adolescents with disabilities: A test of chain mediated effects of self-depletion and self-efficacy. Front. Psychol..

[11] Centers for Disease Control and Prevention (2022). Physical Activity for People with Disability. https://www.cdc.gov/ncbddd/disabilityandhealth/features/physical-activity-for-all.html.

[12] Hardee J.P., Fetters L. (2017). The effect of exercise intervention on daily life activities and social participation in individuals with Down syndrome: A systematic review. Res. Dev. Disabil..

[13] World Health Organization (2020). Guidelines on Physical Activity and Sedentary Behaviour.

[14] Ginis K.A.M., van der Ploeg H.P., Foster C., Lai B., McBride C.B., Ng K., Pratt M., Shirazipour C.H., Smith B., Vásquez P.M. (2021). Participation of people living with disabilities in physical activity: A global perspective. Lancet.

[15] Ramírez A., Figueroa A., Sanz de Gabiña L. (2007). Estudio Sobre los Hábitos Deportivos en las Personas con Discapacidad en la Provincia de Guipuzkoa.

[16] Saebu M. (2010). Physical Disability and Physical Activity: A Review of The Literature on Correlates and Associations. Eur. J. Adapt. Phys. Act..

[17] Boslaugh S.E., Andresen E.M. (2006). Correlates of physical activity for adults with disability. Prev. Chronic Dis..

[18] Bloemen M.A.T., van den Berg-Emons R.J.G., Tuijt M., Nooijen C.F.J., Takken T., Backx F.J.G., Vos M., de Groot J.F. (2019). Physical activity in wheelchair-using youth with spina bifida: An observational study. J. NeuroEng. Rehabil..

[19] Sit C., Aubert S., Carty C., Silva D.A.S., López-Gil J.F., Asunta P., Palad Y., Guisihan R., Lee J., Arbour Nicitopoulos K.P. (2022). Promoting Physical Activity Among Children and Adolescents with Disabilities: The Translation of Policy to Practice Internationally. J. Phys. Act. Health.

[20] Maher C.A., Williams M.T., Olds T., Lane A.E. (2007). Physical and sedentary activity in adolescents with cerebral palsy. Dev. Med. Child Neurol..

[21] Seemüller S., Beck F., Reimers A.K. (2023). Physical activity of children and adolescents who use a wheelchair: A systematic review. BMC Public Health.

[22] Bloemen M.A., Takken T., de Groot J.F., Kruitwagen C.L.J.J., Rook R.A., van den Berg-Emons R.H.J.G., Backx F.J.G. (2020). Determinants of physical activity in young wheelchair-user with spina bifida. J. Rehabil. Med..

[23] Sol M.E., Verschuren O., Horemans H., Westers P., Visser-Meily J.M.A., De Groot J.F., Fit-for-the-Future Consortium (2022). The effects of wheelchair mobility skills and exercise training on physical activity, fitness, skills and confidence in youth using a manual wheelchair. Disabil. Rehabil..

[24] General Authority for Statistics (GASTAT) (2017). APD Statistics, Disability Survey 2017. https://apd.gov.sa/en/.

[25] Al-Hazzaa H.M. (2018). Physical inactivity in Saudi Arabia revisited: A systematic review of inactivity prevalence and perceived barriers to active living. Int. J. Health Sci..

[26] Alqahtani B.A., Alenazi A.M., Alhowimel A.S., Elnaggar R.K. (2021). The descriptive pattern of physical activity in Saudi Arabia: Analysis of national survey data. Int. Health..

[27] AlMarzooqi M.A., Alsukait R.F., Aljuraiban G.S., Alothman S.A., AlAhmed R., Rakic S., Herbst C.H., Al-Hazzaa H.M., Alqahtani S.A. (2023). Comprehensive assessment of physical activity policies and initiatives in Saudi Arabia 2016-2022. Front. Public Health..

[28] Al-Nozha M.M., Al-Hazzaa H.M., Arafah M.R., Al-Khadra A., Al-Mazrou Y.Y., Al-Maatouq M.A., Khan N.B., Al-Marzouki K., Al-Harthi S.S., Abdullah M. (2007). Prevalence of physical activity and inactivity among Saudis aged 30-70 years. A population-based cross-sectional study. Saudi Med. J..

[29] Amin T.T., Al Khoudair A.S., Al Harbi M.A., Al Ali A.R. (2012). Leisure time physical activity in Saudi Arabia: Prevalence, pattern and determining factors. Asian Pac. J. Cancer Prev..

[30] Zahra A., Hassan M.S., Park J.H., Hassan S.U., Parveen N. (2022). Role of Environmental Quality of Life in Physical Activity Status of Individuals with and without Physical Disabilities in Saudi Arabia. Int. J. Environ. Res. Public Health.

[31] Alhumaid M.M., Said M.A., Adnan Y., Khoo S. (2024). Cross-Cultural Adaptation and Validation of the Arabic Version of the Physical Activity Scale for Individuals with Physical Disabilities in Saudi Arabia (PASIPD-AR). Healthcare.

[32] Washburn R.A., Zhu W., McAuley E., Frogley M., Figoni S.F. (2002). The physical activity scale for individuals with physical disabilities: Development and evaluation. Arch. Phys. Med. Rehabil..

[33] Sallis J.F., Saelens B.E. (2000). Assessment of physical activity by self-report: Status, limitations, and future directions. Res. Q. Exerc. Sport..

[34] Tweedy S.M., Trost S.G. (2005). Validity of accelerometry for measurement of activity in people with brain injury. Med. Sci. Sports Exerc..

[35] van der Ploeg H.P., Streppel K.R., van der Beek A.J., van der Woude L.H., Vollenbroek-Hutten M., van Mechelen W. (2007). The Physical Activity Scale for Individuals with Physical Disabilities: Test-retest reliability and comparison with an accelerometer. J. Phys. Act. Health.

[36] Jimenez-Pardo J., Holmes J.D., Jenkins M.E., Johnson A.M. (2005). An Examination of the Reliability and Factor Structure of the Physical Activity Scale for Individuals with Physical Disabilities (PASIPD) Among Individuals Living with Parkinson’s Disease. J. Aging Phys. Act..

[37] van Remoortel H., Camillo C.A., Langer D., Hornikx M., Demeyer H., Burtin C., Decramer M., Gosselink R., Janssens W., Troosters T. (2013). Moderate intense physical activity depends on selected Metabolic Equivalent of Task (MET) cut-off and type of data analysis. PLoS ONE.

[38] World Health Organization (2016). Saudi Arabia Diabetes Country Profiles 2016.

[39] Al-Zalabani A., Al-Hamdan N., Saeed A. (2015). The prevalence of physical activity and its socioeconomic correlates in Kingdom of Saudi Arabia: A cross-sectional population-based national survey. J. Taibah Univ. Med. Sci..

[40] Heath G.W., Levine D. (2022). Physical Activity and Public Health among People with Disabilities: Research Gaps and Recommendations. Int. J. Environ. Res. Public Health.

[41] Carty C., van der Ploeg H.P., Biddle S.J.H., Bull F., Willumsen J., Lee L., Kamenov K., Milton K. (2021). The First Global Physical Activity and Sedentary Behavior Guidelines for People Living with Disability. J. Phys. Act. Health.

[42] Ahmad N.A., Mohamad Kasim N., Mahmud N.A., Mohd Yusof Y., Othman S., Chan Y.Y., Abd Razak M.A., Yusof M., Omar M., Abdul Aziz F.A. (2017). Prevalence and determinants of disability among adults in Malaysia: Results from the National Health and Morbidity Survey (NHMS) 2015. BMC Public Health.

[43] Oyeyemi A.L., Oyeyemi A.Y., Omotara B.A., Lawan A., Akinroye K.K., Adedoyin R.A., Ramírez A. (2018). Physical activity profile of Nigeria: Implications for research, surveillance and policy. Pan Afr. Med. J..

[44] Ellis R., Kosma M., Cardinal B.J., Bauer J.J., McCubbin J.A. (2007). Physical activity beliefs and behaviour of adults with physical disabilities. Disabil. Rehabil..

[45] IPAQ Research Committee (2005). Guidelines for Data Processing and Analysis of the International Physical Activity Questionnaire (IPAQ). https://www.physio-pedia.com/images/c/c7/Quidelines_for_interpreting_the_IPAQ.pdf.

[46] Ganesh G.S., Marwah D., Punyal S., Gupta S. (2020). Physical activity and quality of life predictors among university students with polio in India: A cross-sectional study. J. Clin. Transal. Res..

[47] Winberg C., Flansbjer U.B., Rimmer J.H., Lexell J. (2015). Relationship between physical activity, knee muscle strength, and gait performance in persons with late effects of polio. PM & R..

[48] Van Helm S., Krops L.A., Dekker R., Vrieling A.H. (2022). Effectiveness of (Active) Lifestyle Interventions in People with a Lower Limb Amputation: A Systematic Review. Arch. Rehabil. Res. Clin. Transl..

[49] Davie-Smith F., Coulter E., Kennon B., Wyke S., Paul L. (2017). Factors influencing quality of life following lower limb amputation for peripheral arterial occlusive disease: A systematic review of the literature. Prosthet. Orthot. Int..

[50] van Schaik L., Geertzen J.H.B., Dijkstra P.U., Dekker R. (2019). Metabolic costs of activities of daily living in persons with a lower limb amputation: A systematic review and meta-analysis. PLoS ONE.

[51] Abouammoh N., Aldebeya W., Abuzaid R. (2021). Experiences and needs of patients with lower limb amputation in Saudi Arabia: A qualitative study. East. Mediterr. Health J..

[52] AlSofyani M.A., AlHarthi A.S., Farahat F.M., Abuznadah W.T. (2016). Impact of rehabilitation programs on dependency and functional performance of patients with major lower limb amputations. A retrospective chart review in western Saudi Arabia. Saudi Med. J..

[53] de Hollander E.L., Proper K.I. (2018). Physical activity levels of adults with various physical disabilities. Prev. Med. Rep..

[54] Carver J., Ganus A., Ivey J.M., Plummer T., Eubank A. (2016). The impact of mobility assistive technology devices on participation for individuals with disabilities. Disabil. Rehabil. Assist. Technol..

[55] Scherer M.J., Glueckauf R. (2005). Assessing the Benefits of Assistive Technologies for Activities and Participation. Rehabil. Psychol..

[56] Jutai J., Day H. (2002). Psychosocial impact of assistive devices (PIADS). Technol. Disabil..

[57] Kaye H., Kang T., LaPlante P. (2000). Mobility Device Use in the United States.

[58] Gray D., Quatrano L., Lieberman M., Gray D., Quatrano L., Lieberman M. (1998). Conclusions: Moving to the next stage of assistive technology development. Designing and Using Assistive Technology: The Human Perspective.

[59] Pettee K.K., Brach J.S., Kriska A.M., Boudreau R., Richardson C.R., Colbert L.H., Satterfield S., Visser M., Harris T.B., Ayonayon H.N. (2006). Influence of marital status on physical activity levels among older adults. Med. Sci. Sports Exerc..

[60] Sobal J., Hanson K. (2010). Marital Status and Physical Activity in U.S. Adults. Int. J. Sociol. Fam..

[61] Nomaguchi K.M., Bianchi S.M. (2004). Exercise time: Gender differences in the effects of marriage, parenthood, and employment. J. Marriage Fam..

[62] Thomas P.A., Richards E.A., Forster A.K. (2022). Is Marital Quality Related to Physical Activity Across the Life Course for Men and Women?. J. Aging Health.

[63] Porch T.C., Bell C.N., Bowie J.V., Usher T., Kelly E.A., LaVeist T.A., Thorpe R.J. (2016). The Role of Marital Status in Physical Activity Among African American and White Men. Am. J. Men’s Health.

[64] McAuley E., Zhu W., Chodzko-Zajko W.J. (2006). Physical activity, aging, and quality of life: Implications for measurement. Measurement Issues in Aging and Physical Activity.

[65] Mirowsky J., Ross C.E. (2003). Education, Social Status, and Health.

[66] Said M.A., Alibrahim M. (2022). Physical activity, sedentary behaviors, and breakfast eating as factors influencing BMI in Saudi students, aged 10 to 15 years. Ann. Med..

